# Informing a target product profile for rapid tests to identify HBV-infected pregnant women with high viral loads: a discrete choice experiment with African healthcare workers

**DOI:** 10.1186/s12916-023-02939-y

**Published:** 2023-07-04

**Authors:** Yasir Shitu Isa, Jonathan Sicsic, Henry Njuguna, John Ward, Mohamed Chakroun, Mohamed El-Kassas, Rado Ramanampamonjy, Salim Chalal, Jeanne Perpétue Vincent, Monique Andersson, Hailemichael Desalegn, Fatou Fall, Asgeir Johannessen, Philippa C. Matthews, Gibril Ndow, Edith Okeke, Nicholas Riches, Moussa Seydi, Edford Sinkala, C. Wendy Spearman, Alexander Stockdale, Michael J. Vinikoor, Gilles Wandeler, Roger Sombié, Maud Lemoine, Judith E. Mueller, Yusuke Shimakawa

**Affiliations:** 1 Institut Pasteur, Université Paris Cité, Unité d’Épidémiologie Des Maladies Émergentes, 25-28 Rue du Dr Roux, 75015 Paris, France; 2 EHESP French School of Public Health, Rennes, France; 3 Université Paris Cité, LIRAES F-75006, Paris, France; 4 Coalition for Global Hepatitis Elimination, Decatur, GA USA; 5 Infectious Disease Department, Fattouma Bourguiba Hospital, Monastir, Tunisia; 6 Endemic Medicine Department, Faculty of Medicine, Helwan University, Cairo, Egypt; 7 Unité de Gastro-Entérologie, Hôpital Joseph Raseta Befelatanana, Antananarivo, Madagascar; 8 Plateforme de Data Management, Institut Pasteur, Paris, France; 9 Division of Medical Virology, Stellenbosch University, Faculty of Medicine and Health Sciences, Cape Town, South Africa; 10 Nuffield Division of Clinical Laboratory Science, Radcliffe Department of Medicine, University of Oxford, Oxford, UK; 11 Medical Department, St. Paul’s Hospital Millennium Medical College, Addis Ababa, Ethiopia; 12 Department of Hepatology and Gastroenterology, Hopital Principal de Dakar, Dakar, Senegal; 13 Department of Infectious Diseases, Vestfold Hospital, Tønsberg, Norway; 14 Institute of Clinical Medicine, University of Oslo, Oslo, Norway; 15 The Francis Crick Institute, London, UK; 16 Division of Infection and Immunity, University College London, London, UK; 17 Department of Infectious Diseases, University College London Hospital, London, UK; 18 Medical Research Council Unit The Gambia, London School of Hygiene & Tropical Medicine, Fajara, The Gambia; 19 Department of Metabolism, Digestion & Reproduction, Imperial College London, London, UK; 20 Faculty of Medical Sciences, University of Jos, Jos, Nigeria; 21 Department of Clinical Sciences, Liverpool School of Tropical Medicine, Liverpool, UK; 22 Service de Maladies Infectieuses Et Tropicales, Centre Hospitalier National Universitaire de Fann, Dakar, Senegal; 23 Department of Internal Medicine, University of Zambia, Lusaka, Zambia; 24 Division of Hepatology, Department of Medicine, Faculty of Health Sciences, University of Cape Town, Cape Town, South Africa; 25 Department of Clinical Infection, Microbiology and Immunology, Institute of Infection, University of Liverpool, Liverpool, UK; 26 Malawi-Liverpool-Wellcome Trust Clinical Research Programme, Blantyre, Malawi; 27 Department of Medicine, University of Alabama at Birmingham, Birmingham, AL USA; 28 Department of Infectious Diseases, Bern University Hospital, University of Bern, Bern, Switzerland; 29 Service d’Hépato-Gastroentérologie, Centre Hospitalier Universitaire Yalgado Ouédraogo, Ouagadougou, Burkina Faso

**Keywords:** Hepatitis B, Mother-to-child transmission, Elimination, Rapid diagnostic test, Preferences, Discrete choice experiment, Target product profile, Africa

## Abstract

**Background:**

Elimination of mother-to-child transmission of hepatitis B virus (HBV) requires infant immunoprophylaxis and antiviral prophylaxis for pregnant women with high viral loads. Since real-time polymerase chain reaction (RT-PCR), a gold standard for assessing antiviral eligibility, is neither accessible nor affordable for women living in low-income and middle-income countries (LMICs), rapid diagnostic tests (RDTs) detecting alternative HBV markers may be needed. To inform future development of the target product profile (TPP) for RDTs to identify highly viremic women, we used a discrete choice experiment (DCE) and elicited preference and trade-off of healthcare workers (HCW) in Africa between the following four attributes of fictional RDTs: price, time-to-result, diagnostic sensitivity, and specificity.

**Methods:**

Through an online questionnaire survey, we asked participants to indicate their preferred test from a set of two RDTs in seven choice tasks with varying levels of the four attributes. We used mixed multinomial logit models to quantify the utility gain or loss generated by each attribute. We attempted to define minimal and optimal criteria for test attributes that can satisfy ≥ 70% and ≥ 90% of HCWs, respectively, as an alternative to RT-PCR.

**Results:**

A total of 555 HCWs from 41 African countries participated. Increases in sensitivity and specificity generated significant utility and increases in cost and time-to-result generated significant disutility. The size of the coefficients for the highest attribute levels relative to the reference levels were in the following order: sensitivity (β = 3.749), cost (β = -2.550), specificity (β = 1.134), and time-to-result (β = -0.284). Doctors cared most about test sensitivity, while public health practitioners cared about cost and midwives about time-to-result. For an RDT with 95% specificity, costing 1 US$, and yielding results in 20 min, the minimally acceptable test sensitivity would be 82.5% and the optimally acceptable sensitivity would be 87.5%.

**Conclusions:**

African HCWs would prefer an RDT with the following order of priority: higher sensitivity, lower cost, higher specificity, and shorter time-to-result. The development and optimization of RDTs that can meet the criteria are urgently needed to scale up the prevention of HBV mother-to-child transmission in LMICs.

**Supplementary Information:**

The online version contains supplementary material available at 10.1186/s12916-023-02939-y.

## Background

Hepatitis B virus (HBV) represents a major global health burden with 296 million people living with chronic HBV infection (CHB) [1]. Low-income and middle-income countries (LMICs) are disproportionately affected, with the African region having an estimated 6.5% prevalence of CHB [2]. WHO has developed 2030 elimination targets and one of its main objectives is to reduce the incidence of CHB by 90% [1, 3].

In countries with high CHB prevalence, mother-to-child transmission (MTCT) remains the major transmission route and linked to high likelihood of developing chronic infection and severe liver disease [4, 5]. To prevent MTCT, WHO recommends that all infants should receive three doses of HBV vaccine starting within 24 h of birth [6]. However, the infant immunoprophylaxis does not prevent all MTCT particularly when infected mothers have high viral loads [7]. Consequently in 2020 WHO additionally recommended that all pregnant women found to carry hepatitis B surface antigen (HBsAg) should undergo nucleic acid testing (NAT) to quantify serum HBV DNA levels, and those identified to have high viral loads (≥ 200,000 IU/ml) should initiate prophylactic antivirals [6].

Commercially-available NAT using real-time polymerase chain reaction (RT-PCR), however, is expensive (US$ 20–130/test) and not widely available in LMICs [8, 9]. Therefore, in the 2020 guidelines, WHO conditionally recommended the use of hepatitis B e antigen (HBeAg) as an alternative to HBV DNA quantification where RT-PCR is not available [6]. HBeAg is a classical serological marker of viral replication and detected by both laboratory-based immunoassay and immunochromatographic rapid diagnostic test (RDT) [10]. Similarly, other serological markers for HBV, such as quantification of HBsAg or hepatitis B core-related antigen (HBcrAg), might be also helpful to identify high-risk pregnant women in LMICs, because of their close correlations with serum HBV DNA levels and their potential applications to an immunochromatography assay [11–13]. Despite the advent of these promising technologies, there is still no “Target Product Profile (TPP)” outlining the necessary features of diagnostic tools adapted to LMICs to identify pregnant women with high HBV DNA levels [14]. TPP is useful in guiding manufacturers to develop and optimize new tests, particularly for LMICs [14].

It would be ideal to have an RDT that costs as low as US$ 1, providing results in < 30 min, with 100% sensitivity and 100% specificity to identify highly viremic women (≥ 200,000 IU/ml). However, developing such a test within a short time horizon is unlikely; stakeholders have to accept some trade-off and arbitrate between these characteristics. We, therefore, conducted a discrete choice experiment (DCE) to assess the preference of healthcare workers (HCWs) in Africa on the following four characteristics of fictional RDTs to identify HBV-infected pregnant women eligible for prophylactic antivirals: price of test paid by the woman, time-to-result, diagnostic sensitivity, and diagnostic specificity. We then attempted to define “minimal” and “optimal” criteria for these parameters in order to inform future TPP for RDTs to diagnose high HBV DNA levels.

## Methods

### Study design

DCE is a well-established quantitative method used to elicit stated preferences between hypothetical alternative scenarios [15]. Briefly, DCE aims to understand what people prefer when faced with different choices or options. In a DCE, individuals are asked to choose between hypothetical alternative scenarios, goods or services, each described by several attributes, such as price, quality, or availability. The responses are used to determine which attributes are most important to people when making choices, and how much they value each attribute. This information can be used to design policies or services that better meet the needs and preferences of patients and health professionals. While DCE has been commonly used in high-income countries, this is increasingly being applied in LMICs to address a range of health policy concerns [16].

**Fig. 1:**
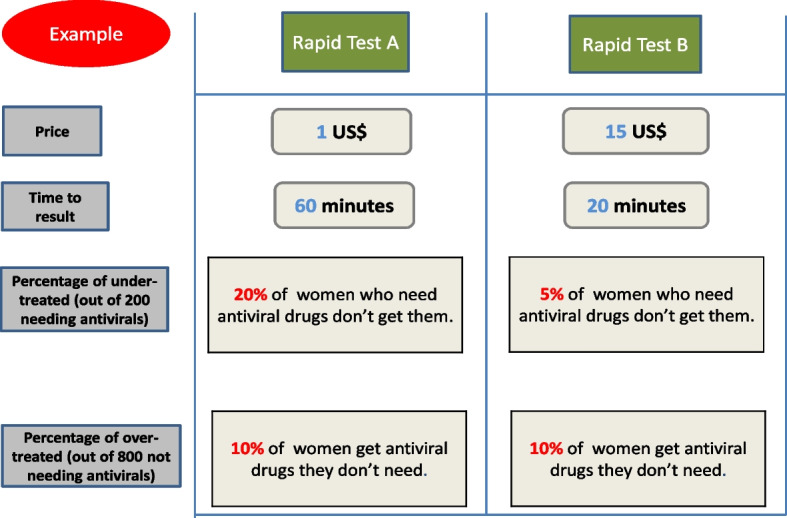
Example of choice task. Respondents were asked to choose their preferred rapid diagnostic test (RDT)

We conducted a DCE survey using a self-administered online questionnaire and asked participants to repeatedly indicate their preferred test from a set of two fictional alternatives (test A and test B) offering a unique combination of the four characteristics/attributes (Fig. 1). The questionnaire consisted of four parts: i) the rationale for the survey describing an unmet medical need for the prevention of HBV MTCT and the challenges of accessing RT-PCR in LMICs; ii) a detailed description of the context in which the choices should be made; iii) a DCE questionnaire with seven choice tasks; and iv) a short questionnaire about survey respondents (Additional file 1). We first produced the questionnaire in English, then obtained the French and Portuguese versions through official translators (Additional file 1).

### Participants

All types of HCWs practicing in Africa (doctors, nurses, midwives, laboratory workers, and public health practitioners) were eligible for the study, irrespective of their experience of caring for people infected with HBV. We sent the invitation via email to HCWs listed in an existing database of stakeholders involved in previous hepatitis B projects. We also sought the collaboration of the Coalition for Global Hepatitis Elimination (CGHE) and Hepatitis B in Africa Collaborative Network (HEPSANET) to pilot the questionnaire and to help disseminate the survey across the continent. We used the chain-referral sampling technique. Study participation was entirely anonymous and no informed consent was required. The Institutional Review Board at the Institut Pasteur formally exempted the protocol from a full review (Reference: IRB2021-I-Exempt). The survey platform was open from March 25 to May 13, 2022.

### DCE tool development

#### Choice of attributes and levels

Firstly, to define the attributes we reviewed the literature on hepatitis diagnostics, including the WHO’s TPP for the diagnosis of hepatitis C viremia [17]. To set the range of attribute levels, we referred to the commercially-available reference RT-PCR (US$40, time-to-result ≥ 60 min, sensitivity 100%, specificity 100%) and commercially-available rapid HBeAg tests (US$ 0.5–1.3, time-to-result 15–20 min, sensitivity 67–77%, and specificity 95–97%) [3, 18–22]. Secondly, we held in-depth discussion with experts in hepatitis B management in LMICs (clinicians, epidemiologists, health economists, health policy experts) to refine the attributes and their levels. Finally, we piloted the draft DCE questionnaire with six African HCWs and subsequently interviewed them using think-aloud technique to understand their information processing pattern and thoughts [23, 24]. As healthcare workers may misinterpret the terms “sensitivity” and “specificity”, [25] we carefully replaced these by “percentage of under-diagnosis/under-treatment” and “percentage of over-diagnosis/over-treatment”, respectively. Table 1 presents the final list of attributes and their levels.

**Table 1:** Attributes and levels

Attributes	Description	Levels
**Price payable by women / test**	Cost of the test borne by the HBV-infected pregnant woman	1-US$ 1
		2-US$ 5
		3-US$ 15
		4-US$ 20
**Time-to-result**	Time to process the test and provide results	1–20 min
		2–60 min
**Diagnostic sensitivity**	Probability of the RDT to give a positive result if woman has high viral load (≥ 200,000 IU/mL). To ease interpretation, this attribute was presented as the percentage of under-diagnosed women	1–85% (15% of women under-treated)
		2–90% (10% of women under-treated)
		3–95% (5% of women under-treated)
		4–100% (0% of women under-treated)
**Diagnostic specificity**	Probability of the RDT to give a negative result if woman has low viral load (< 200,000 IU/mL). To ease interpretation, this attribute was presented as the percentage of over-diagnosed women	1–90% (10% of women over-treated)
		2–95% (5% of women over-treated)
		3–100% (0% of women over-treated)

#### Experimental design

Having four price levels, two time-to-result levels, four sensitivity levels, and three specificity levels, would allow the construction of 96 fictional RDTs using a full factorial design. We selected the relevant combinations of the attributes using STATA 17.0 (Stata Corporation, College Station, TX), by assigning pseudo-informative priors based on a priori assumptions (Additional file 2). We determined that a minimum of 12 choice tasks were needed to obtain estimates for inferring preference. Since completing 12 choice tasks would take > 30 min for a participant, we made two blocks of six choice tasks and randomly allocated participants to one of these blocks. In addition, to evaluate the monotonicity of responses, we conducted a “dominance test” by adding in each block a seventh choice task, where one test had better levels in all four attributes than the other. We considered respondents as “rational” if they chose the test in which all attributes’ levels are better than the other and “non-rational” if they chose the test in which all attribute’s’ levels are worse than the other.

### Statistical analysis

We described participant characteristics based on dominant test result and evaluated whether the response pattern differed between non-rational and rational responders by comparing the proportion choosing “test A” in each choice task using a chi-squared test. We analyzed the choice data within a random utility maximization framework [26]. We first used the alternative-specific multinomial (ASM) probit regression which has short computation time and allows estimation of average preference estimates and investigation of observed preference heterogeneity through interaction analysis. For the attributes having ≥ 3 levels, we examined the linear relationship between their levels and the coefficient values using a scatter diagram and fitting a regression line. Once we confirmed the linearity, we converted the attribute levels from categorical to continuous. We explored the interactions between individual characteristics and test attributes using continuous attribute levels. For the main analysis, we used mixed multinomial logit (MIXL), which is computationally intense but allows the preference parameters to be randomly distributed across the sample and thus accounting for unobserved preference heterogeneity and correlation of choices within participants. We added an alternative specific constant (ASC) for test A to assess the propensity to select test A versus test B irrespective of their attributes’ levels (Additional file 3) [27–29].

To define the “minimal” and “optimal” TPP criteria for the combination of attributes, we computed the probability of preferring an RDT with specific profiles to RT-PCR through utility modelling using MIXL coefficients of continuous attributes. We first determined the utility of each fictional RDT by summing a loss or gain attributed to each of the four attributes of this specific test. We then used the predictive formula below to estimate the probability of preferring this RDT to RT-PCR by comparing its utility with that of RT-PCR.

$${U}_{test} =\mathrm{ \alpha }ASC\mathrm{test}A + \{\beta \mathrm{Cost}*(\mathrm{Cost}-1)\} + \{\beta \mathrm{Sensitivity}*(\mathrm{Sensitivity}-85)\}+ \{\beta \mathrm{Specificity}*(\mathrm{Specificity}-90)\}+ \{\beta \mathrm{Time}*(\mathrm{Time}-20)/40)\}$$

$$\mathrm{Probability of preferring the RDT to RT-PCR}= \frac{{\mathrm{exp}}^{{\mathrm{U}}_{\mathrm{test}}}}{{\mathrm{exp}}^{{\mathrm{U}}_{\mathrm{test}}}+{\mathrm{exp}}^{{\mathrm{U}}_{\mathrm{pcr}}}} * 100$$

where $${U}_{pcr}$$ denotes the utility associated with the RT-PCR with the following attribute values: cost = US$40, time-to-result = 60 min, sensitivity = 100%, specificity = 100%. After the discussion with the experts in the field, we applied the probability threshold of ≥ 70% and ≥ 90% to identify a set of characteristics meeting a “minimal” and “optimal” TPP of RDTs, respectively, to diagnose HBV-infected pregnant women eligible for antiviral prophylaxis in LMICs.

## Results

A total of 576 HCWs completed the online survey; after excluding 21 from outside Africa, the analysis included 555 HCWs from 41 African countries. Table 2 presents their characteristics. The majority (70.6%) were between 30 and 50 years old and 44.5% were females. They were doctors (62.9%), public health practitioners (10.3%), laboratory staff (9.4%), midwives (5.6%), and nurses (4.1%). Their place of work was national hospital (28.5%), provincial hospital (12.8%), district hospital (10.3%), primary care (7.7%), private (11.7%), public health (18.6%), and other (10.4%). More than half (66.3%) reported being involved in either providing HBV patient management or programs. Overall, 69.2%, 9.5%, 9.4%, 6.7%, and 5.2% worked in West, Southern, Eastern, Northern, and Central Africa, respectively. In the dominance tests, most participants (85.9%, 477/555) were rational responders. Rational responses were more frequently observed in women than in men (89.9% vs 82.8%), and in doctors than in nurses (89.4% vs 73.9%) (Table 2). In half of the 12 “non-dominant” choice tasks, the pattern of the choice between two alternatives was significantly different between the rational and non-rational responders (Additional file 4: Table S1).

**Table 2:** Participant characteristics and their association with being rational responders

	**Full sample**		**Rational responders**		**Non-rational responders**		
	***n*** ** = 555**	**%**	***n*** ** = 477**	**%**	***n*** ** = 78**	**%**	***P*** **-value**
**Age group (years)**							
< 30	78	14.1	64	82.0	14	18.0	0.486
30–50	392	70.6	341	87.0	51	13.0	
> 50	85	15.3	72	84.7	13	15.3	
**Gender**							
Male	308	55.5	255	82.8	53	17.2	0.017
Female	247	44.5	222	89.9	25	10.1	
**Profession**							
Doctor	349	62.9	312	89.4	37	10.6	0.030
Nurse	23	4.1	17	73.9	6	26.1	
Midwife	31	5.6	24	77.4	7	22.6	
Laboratory staff	52	9.4	45	86.5	7	13.5	
Public health practitioner	57	10.3	46	80.7	11	19.3	
Other	43	7.7	33	76.7	10	23.3	
**Sector**							
Public/Primary Care	43	7.7	31	72.1	12	27.9	0.126
Public/District Hospital	57	10.3	49	86.0	8	14.0	
Public/Provincial Hospital	71	12.8	63	88.7	8	11.3	
Public/National Hospital	158	28.5	141	89.2	17	10.8	
Private	65	11.7	58	89.2	7	10.8	
Public health sector	103	18.6	87	84.5	16	15.5	
Other	58	10.4	48	82.8	10	17.2	
**Region (Africa)**							
North	37	6.7	33	89.2	4	10.8	0.453
Central	29	5.2	22	75.9	7	24.1	
East	52	9.4	47	90.4	5	9.6	
South	53	9.5	46	86.8	7	13.2	
West	384	69.2	477	86.0	78	14.0	
**Hepatitis B care involvement**							
No	187	33.7	162	86.6	25	13.4	0.741
Yes	368	66.3	315	85.6	53	14.4	

### Alternative-specific multinomial (ASM) probit regression analysis

In the ASM probit regression model including all respondents (n = 555), all attribute levels, except the time-to-result of 60 min, had statistically significant coefficients (Additional file 4: Table S2). The increase in cost was significantly associated with a decrease in the coefficients, while the increase in sensitivity and specificity was associated with an increase in the coefficients. The size of the coefficients (β) for the highest attribute levels relative to the reference levels were in the following order: sensitivity (1.461), cost (-1.118), specificity (0.522), and time-to-result (-0.031). When the model contained only rational responders (n = 477), all coefficients of the eight attribute levels remained statistically significant with an increase in their absolute values compared to the analysis including all responders (Additional file 4: Table S2). This suggested the presence of non-differential misclassification among non-rational responders; consequently, we excluded them from the subsequent analyses. Given the high level of linear correlation between the attribute levels and the coefficient values (Additional file 4: Figure S1), we subsequently considered cost, sensitivity, and specificity as continuous variables. Additional file 4: Table S3 presents the results of the ASM probit with continuous variables.

### Mixed multinomial logit (MIXL) models

In our main analysis with continuous variables in 477 rational responders, all attribute levels had statistically significant coefficients (Table 3). A unit increase in sensitivity and specificity generated significant positive utilities (β = 0.269 and 0.132, respectively) while unit increase in cost and time-to-result induced significant utility loss (β = -0.154 and -0.174, respectively). To evaluate the propensity to select test A versus test B irrespective of the attribute levels, we added ASC for test A (Table 3). In the MIXL model containing all responders (n = 555), there was an evidence to support the systematic propensity to choose test A versus test B (*β*testA = 0.305, *p* < 0.001). In contrast, among the rational responders (n = 477), there was no systematic propensity to choose test A (*β*testA = -0.026, p = 0.758). This indicated that rational responders could have effectively made a trade-off between the two test alternatives whilst non-rational responders could not, again supporting the exclusion of the latter group from the main analysis.

**Table 3:** Mixed multinomial logit (MIXL) model with continuous attribute levels

		**Full sample (** ***n*** ** = 555)**				
	Mean			Standard deviation		
	Coefficient	Standard error	*P*-value	Coefficient	Standard error	*P*-value
ASC: Test A	0.305	0.085	< 0.001	1.217	0.119	< 0.001
Attributes						
Cost (per US$1 increase)	-0.133	0.010	< 0.001	0.108	0.010	< 0.001
Sensitivity (per 1% increase)	0.221	0.017	< 0.001	0.223	0.019	< 0.001
Specificity (per 1% increase)	0.115	0.012	< 0.001	-0.039	0.027	0.150
Time (from 20 to 60 min)	-0.173	0.065	0.008	0.519	0.145	< 0.001
		**Rational responders (** ***n*** ** = 477)**				
	Mean			Standard deviation		
	Coefficient	Standard error	*P*-value	Coefficient	Standard error	*P*-value
ASC: Test A	-0.026	0.085	0.758	0.928	0.128	< 0.001
Attributes						
Cost (per US$1 increase)	-0.154	0.012	< 0.001	0.107	0.012	< 0.001
Sensitivity (per 1% increase)	0.269	0.021	< 0.001	0.241	0.021	< 0.001
Specificity (per 1% increase)	0.132	0.014	< 0.001	0.059	0.027	0.005
Time (from 20 to 60 min)	-0.174	0.073	0.042	0.603	0.144	< 0.001

ASC denotes alternative specific constant for test A

Additional file 4: Table S4 presents the results of the MIXL models with categorical variables in the rational responders. As has been observed for the ASM probit regression analysis, the coefficient size (β) for the highest attribute levels relative to the reference levels was in the following order: sensitivity (3.749), cost (-2.550), specificity (1.134), and time-to-result (-0.284).

### “Minimal” and “optimal” TPP

**Fig. 2:**
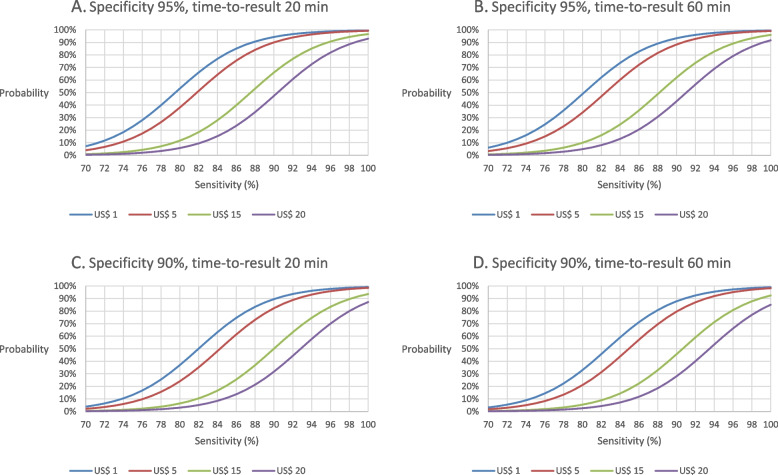
Probability of preferring an RDT to RT-PCR. **A**. Probability of preferring an RDT with a specificity of 95% and a time-to-result of 20 min. **B**. Probability of preferring an RDT with a specificity of 95% and a time-to-result of 60 min. **C**. Probability of preferring an RDT with a specificity of 90% and a time-to-result of 20 min. **D**. Probability of preferring an RDT with a specificity of 90% and a time-to-result of 60 min

Figure 2 presents the probability of preferring an RDT to RT-PCR by varying the specificity between 90 and 95%, time-to-result between 20 and 60 min, cost from US$ 1 to 20, and sensitivity from 70 to 100%. Assuming a specificity of 95% and a time-to-result of 20 min (Fig. 2A), the minimally acceptable threshold for sensitivity that can satisfy ≥ 70% of African HCWs was 82.5%, 85.0%, 90.5%, and 93.5%, when the test costs US$1, 5, 15, and 20, respectively; the optimally acceptable threshold that can satisfy ≥ 90% of HCWs was 87.5%, 90.0%, 95.5%, and 98.5%, respectively (Additional file 4: Table S5). Assuming a specificity of 90% and a time-to-result of 60 min (Fig. 2D), the minimally acceptable threshold for diagnostic sensitivity was 85.5%, 88.0%, 93.5%, and 96.5%, respectively, and the optimally acceptable threshold was 91.5%, 93.0%, 98.5%, and 100%, respectively, when the test costs US$1, 5, 15, and 20.

### Interaction

Table 4 presents the interaction between participant characteristics and test attributes using the ASM probit regression analysis. Compared to young age group, older participants had a greater loss of utility for an increase in price and a smaller gain in utility for an increase in sensitivity. Regarding the profession type, loss of utility for an increase in cost was greater in public health practitioners (β = -0.101) than doctors (-0.067) or midwives (-0.043). By contrast, gain in utility for an increase in sensitivity was higher in doctors (0.129) than in nurses (0.074) or public health practitioners (0.070). Increasing the time-to-result from 20 to 60 min substantially reduced utility for midwives (-0.505) but not much for doctors (-0.011). Heterogeneity in preference was also observed across the working places and across the sub-regions of Africa.

**Table 4:** Interaction between unit increase in test attributes and participants characteristics among rational responders (*n* = 477)

**Characteristics**		**Test attributes**			
		**Cost utility per US$1 increase**	**Sensitivity utility per 1% increase**	**Specificity utility per 1% increase**	**Time utility from 20 to 60 min**
**Age group (years)**					
< 30	**Ref**	**-0.039**	**0.153**	**0.077**	**0.030**
30–50		-0.069^***^	0.115^***^	0.056	-0.056
> 50		-0.090^***^	0.075^***^	0.045^*^	0.057
**Profession**					
Doctor	**Ref**	**-0.067**	**0.129**	**0.053**	**-0.011**
Nurse		-0.046	0.074^**^	0.070	0.287
Midwife		-0.043^*^	0.099	0.103^**^	-0.505^***^
Lab staff		-0.066	0.104	0.044	-0.079
Public health practitioner		-0.101^***^	0.070^***^	0.065	-0.074
Other		-0.067	0.084^***^	0.072	0.129
**Sector**					
Public/National Hospital	**Ref**	**-0.061**	**0.145**	**0.050**	**0.002**
Public/Primary Care		-0.055	0.086^***^	0.103^**^	-0.322^**^
Public/District Hospital		-0.051	0.113^*^	0.088^**^	-0.159
Public/Provincial Hospital		-0.075	0.120	0.038	0.061
Private		-0.054	0.137	0.054	-0.045
Public health sector		-0.081^**^	0.086^***^	0.054	0.042
Other		-0.094^***^	0.072^***^	0.054	0.002
**Region (Africa)**					
North	**Ref**	**-0.042**	**0.192**	**0.052**	**0.035**
Central		-0.064	0.104^***^	0.053	-0.087
East		-0.081^***^	0.073^***^	0.061	0.032
South		-0.063	0.111^***^	0.080	-0.007
West		-0.069^**^	0.116^***^	0.055	-0.040

Statistical significance compared to the reference category

^*^ *p* < 0.1, ** *p* < 0.05, *** *p* < 0.01

## Discussion

To our knowledge, this is the first DCE survey eliciting African HCWs’ trade-offs between the test performance, price, and time-to-result of an RDT to identify HBV-infected pregnant women with a high risk of MTCT in areas with limited access to RT-PCR. By administering a pre-piloted questionnaire translated into three languages to more than 500 participants across the region, we found that all of these parameters had a significant impact on their choice, with the following order of priority: higher sensitivity, lower cost, higher specificity, and shorter time-to-result. Using the utility obtained by the DCE, we also defined “minimal” and “optimal” criteria in order to inform future TPP for RDTs to diagnose high HBV viral loads.

In stated preferences surveys, it is known that respondents may use decision heuristics or mental shortcuts to facilitate the decision process [30, 31]. Indeed, in our survey 14.1% of participants chose a test which had worse levels in all the four attributes; their pattern of the choice significantly differed from that of the “rational responders” and there was a significant propensity in this group to select “test A” irrespective of the attribute levels. To ensure the accuracy of the survey responses, we excluded non-rational responders from the main analyses. Of note, their exclusion resulted in an increase in absolute value of the coefficients in both ASM probit and MIXL models, which strongly suggests the presence of non-differential misclassification among non-rational responders probably due to their random responses or non-understanding of the exercise.

Unsurprisingly, we found that the HCWs prefer a test with higher sensitivity, higher specificity, lower price, and shorter time-to-result. Of these parameters, the test sensitivity was the most important attribute to HCWs, as indicated by the highest value of its coefficient within a realistic range of attribute levels (Additional file 4: Tables S2 & S4). The finding of a strong preference for test sensitivity is consistent with previous DCE works assessing HCWs’ preference for diagnostic tests [32–35]. Importantly, the interaction analysis revealed a striking variation in their trade-offs across the profession type; doctors care most about test sensitivity, while public health practitioners care about cost and midwives about the time it takes to get results for their clients. Similarly, how people make trade-offs between cost and sensitivity differed depending on where they work and where they are geographically located; those working in a referral hospital or private facilities care most about high sensitivity and not low cost, and vice versa for those in primary care or public health. Participants from North Africa are most concerned about high sensitivity and the least about the cost compared to participants from the rest of Africa. Such heterogeneity could be explained at least in part by differences in healthcare resources between settings.

Our work aims to contribute to the scaling up of HBV MTCT prevention programs in LMICs by facilitating the development of a TPP of RDTs to identify pregnant women eligible for antiviral prophylaxis. TPP, a tool defining the necessary features of an innovative product to meet an unmet medical need, has been useful in guiding manufacturers to develop and optimize new tests, particularly for LMICs [14]. However, a recent systematic review of TPP development methodology identified important limitations, such as a lack of transparency in methodology reporting, lack of focus on the trade-off between cost and patient benefit, and subjectivity of information sources [14]. Indeed, 73% of previous TPPs relied on expert opinions to define desirable features and they often provide the optimal and ideal price without considering the trade-off with other parameters [14]. By using the DCE survey targeting local service providers, we attempted to define a minimal and optimal criteria for the combination of test characteristics that can satisfy ≥ 70% and ≥ 90% of HCWs, respectively, as an alternative to the reference RT-PCR test. We believe that this work provides a good example of an application of DCE that may inform the future development of “evidence-based” TPP.

In 2020, WHO made a conditional recommendation on the use of HBeAg test based on the results of a systematic review and meta-analysis; pooled sensitivity and specificity to detect HBV DNA levels ≥ 200,000 IU/mL in pregnant women were 88.2% and 92.6%, respectively [18]. However, these estimates were mostly based on a laboratory-based immunoassay; by restricting the analyses to those using RDT to detect HBeAg, the specificity was 95.7% but the sensitivity was only 70.1% [18, 20, 22]. Assuming that HBeAg RDT costs US$ 1 and provides a result in 20 min, only 10% of HCWs would choose HBeAg RDT over RT-PCR (Fig. 1A). Improvement in its performance or the use of other antigens, such as HBcrAg, [36] will be required for an RDT to be preferred to RT-PCR by the majority of African HCWs.

The study has limitations. First, we did not elicit the preference of pregnant women themselves. In high-income countries, DCE surveys targeting both HCWs and pregnant women for prenatal tests to diagnose genetic disorders revealed that while HCWs preferred higher accuracy, pregnant women attached higher value to the safety of the test procedure [34, 35]. Understanding the willingness of pregnant women to pay for the test should be assessed in the future. Second, the use of online survey may have reduced the generalizability of the results, in particular to HCWs with limited access to internet and email services. In addition, although we had a large sample size from 41 African countries representing five sub-regions, the respondents were still diverse within those sub-regions, with potentially significant differences in healthcare systems, resources, and workforce. As such, caution should be taken when generalizing our findings beyond the study population. Third, the DCE survey is based on stated preference with theoretical choices; respondents could make different choices in real-life situations. Finally, TPP provides a comprehensive picture of required test characteristics that go far beyond the four attributes that we have assessed; these include, but are not limited to, sample type, test procedure, storage conditions, shelf life, and so on [14].

## Conclusions

African HCWs would prefer an RDT with the following order of priority: higher sensitivity, lower cost, higher specificity, and shorter time-to-result. The development and optimization of RDTs that can meet the criteria identified in this DCE work are urgently needed to scale up the prevention of HBV mother-to-child transmission in LMICs, that is a key intervention to achieve global hepatitis elimination.

## Supplementary Information

**Additional file 1. **Survey questionnaire.

**Additional file 2. **Experimental design.

**Additional file 3. **Mixed multinomial logitmodel.

**Additional file 4: Table S1.** Description of choice tasks and choice pattern between rational and non-rational responders. **Table S2.** Alternative-specific multinomial probit regression model with categorical attribute levels. **Table S3.** Alternative-specific multinomial probit regression model with continuous attribute levels. **Table S4.** Mixed multinomial logitmodel with categorical attribute levels. **Table S5.** Minimal and optimal target product profile. **Figure S1.** Scatter plots showing the correlation between the levels of cost, sensitivity and specificity, and the corresponding values for the utility in the rational responders.

## Data Availability

The datasets used and analysed during the current study are available from the corresponding author on reasonable request.

## Abbreviations

ASC: Alternative specific constant
ASM: Alternative-specific multinomial
CGHE: Coalition for Global Hepatitis Elimination
CHB: Chronic HBV infection
DCE: Discrete choice experiment
HBcrAg: Hepatitis B core-related antigen
HBeAg: Hepatitis B e antigen
HBsAg: Hepatitis B surface antigen
HBV: Hepatitis B virus
HCWs: Healthcare workers
HEPSANET: Hepatitis B in Africa Collaborative Network
LMICs: Low-income and middle-income countries
MIXL: Mixed multinomial logit
MTCT: Mother-to-child transmission
NAT: Nucleic acid testing
RDT: Rapid diagnostic test
RT-PCR: Real-time polymerase chain reaction
TPP: Target Product Profile
