# Supplementary material for: Informing a target product profile for rapid tests to identify HBV-infected pregnant women with high viral loads: a discrete choice experiment with African healthcare workers
Source: BMC Med. 2023 Jul 4;21:243. doi: 10.1186/s12916-023-02939-y (PMC10320875; doi:10.1186/s12916-023-02939-y)
Supplement: Supplementary file 1 — Additional file 1. Survey questionnaire. [file 12916_2023_2939_MOESM1_ESM.docx]

**Additional file 1. Survey questionnaire**

**1A. English version**

**Section 1: Introduction**

We invite you to participate in this online survey about your preference on characteristics of a fictional rapid diagnostic test to identify HBV-infected pregnant women with a high risk of mother-to-child transmission.

- To complement the Hepatitis B vaccine birth dose in preventing mother-to-child transmission of hepatitis B, the WHO recommends:

All pregnant women should have a screening test for HBV infection.

All women positive for HBV screening test should further undergo the *PCR test** to quantify hepatitis B viral loads.

All women with a *high viral load*** by PCR test should receive antiviral drugs.

- But, in many low-income countries, HBV-infected women DO NOT benefit from antiviral drugs because:

PCR is NOT available in primary care clinics

Women have to pay out of pocket and CANNOT afford the high price of PCR (US$ > 40/test)

- Therefore, it will be useful to have a rapid test to distinguish between “*high*” and “*low*” viral loads:

At a lower cost affordable for women

Using a drop of blood from the finger

By non-laboratory workers (doctors, nurses, midwives) in primary care clinics

Within a short period of time

- But, as a downside of a rapid test, results may not be as accurate as PCR:

With a risk of over-diagnosis, incorrectly identifying women as having a *high viral load* when their viral load is *low*. This results in over-treatment.

With a risk of under-diagnosis, incorrectly identifying women as having a *low viral load* when their viral load is *high*. This results in under-treatment.

- In this questionnaire, we would like to identify **acceptable trade-offs** between:

The test performance (percentage over-diagnosed / under-diagnosed),

The out-of-pocket cost of the test for women, and

The time-to-result.

* Polymerase Chain Reaction (PCR) assay is a type of nucleic acid testing.

** High viral loads are defined as HBV DNA levels ≥ 200 000 IU/mL.

**Section 2: Example**

Before answering the questions, please imagine that:

- You are a healthcare worker in charge of antenatal care in a primary care clinic in a rural area of low-income country. Most people are very poor living below US$ 1.9/day.

- In each year, you identify about 1000 women infected with HBV at your antenatal screening services.

Of these HBV-infected women, about 20% (200 women) have high viral loads and require antiviral drugs to stop the transmission of HBV to their babies.

The rest (800 women) have low viral loads and do not require antiviral drugs.

However, most of women do not benefit from antiviral drugs because they cannot afford to bear the cost of PCR (US$ 40) and make a one-day trip to a district hospital for PCR.

- You are now asked about your preference between two fictional rapid tests as a tool to detect HBV-infected pregnant women with high viral loads.

- Test A is far more affordable (US$ 1) than Test B (US$ 15) for the women.

- Time to result is longer with Test A (60 min) than Test B (20 min).

- Out of 200 women who need treatment, 20% will be missed using Test A. By using Test B, 5% will be missed.

- Out of 800 women who do not need treatment, 10% will be unnecessarily treated using test A. The same percentage of women will be unnecessarily treated using Test B.

- After choosing one of the rapid tests, you will be asked whether you would accept this rapid test over the conventional PCR. With PCR, there is a risk of lost to follow-up of women due to cost of test and travelling.

**Section 3: The questionnaire**

At first, you will have seven (7) scenarios in which you will be asked to choose between two different rapid tests at each time. Then, you will be asked about your country and job.

Thank you in advance for completing this questionnaire.

**Question on choice task scenarios**

1. Which rapid test do you prefer?

Rapid Test A

Rapid Test B

2. Would you accept this rapid test over PCR*?

YES

NO

* By choosing PCR, woman may drop out as she has to make a one-day trip to a district hospital and bear the cost of PCR (US$ 40).

**** If a participant chooses NO in question 2 for all choice tasks,**

3. Could you state the main reasons why you would never accept a rapid test over a PCR?

PCR has better performance

Other (please specify……………)

***Please click your choice for each task***

**Question about yourself**

Q1: What is your age range?

< 30 years old

30-50 years old

> 50 years old

Q2: What is your gender?

Woman

Man

Other

Q3: In which country do you work?

………………………………

Q4: What is your profession?

Medical doctor/physician

Nurse

Midwife

Laboratory staff

Public health practitioner

Other (please specify ________)

Q5: In what sector do you work?

Healthcare in public sector

Primary care

District hospital

Provincial/Regional hospital

National hospital

Healthcare in private sector

Other (please specify _______)

Q6: Are you involved in hepatitis care?

No

Yes

**1B. French version**

**Section 1: Introduction**

Nous vous invitons à participer à cette enquête en ligne sur votre préférence concernant les caractéristiques d'un test de diagnostic rapide fictif pour identifier les femmes enceintes infectées par le VHB présentant un risque élevé de transmission mère-enfant.

- Pour prévenir la transmission mère-enfant de l'hépatite B, l'OMS recommande :

Toutes les femmes enceintes devraient passer un test de dépistage de l'infection par le VHB.

Toutes les femmes positives au test de dépistage du VHB doivent en plus faire le test PCR* pour quantifier la charge virale de l'hépatite B.

Toutes les femmes ayant une *charge virale élevée*** suite au test PCR doivent recevoir des médicaments antiviraux.

- Mais, dans de nombreux pays à faible revenu, les femmes infectées par le VHB NE bénéficient PAS des médicaments antiviraux car :

La PCR n'est PAS disponible dans les centres de santé.

Les femmes doivent payer de leur poche et NE PEUVENT PAS se permettre le coût élevé de la PCR (US$ > 40/test)

- Par conséquent, il sera utile d'avoir un test rapide pour faire la distinction entre les charges virales « *élevées*» et « *basses* » :

A moindre coût, plus abordable pour les femmes

En utilisant une goutte de sang du bout du doigt

Effectué par les personnels soignant non-rattaché aux laboratoires (médecins, infirmières, sages-femmes) dans les centres de santé.

Dans un court laps de temps

- Mais, comme inconvénient, les résultats d’un test rapide peuvent ne pas être aussi précis que la PCR :

Avec un risque de sur-diagnostic, en identifiant incorrectement des femmes comme ayant une *charge virale élevée* alors que leur charge virale est *basse*. Il en résulte un sur-traitement.

Avec un risque de sous-diagnostic, en identifiant incorrectement des femmes comme ayant une *charge virale basse* alors que leur charge virale est *élevée*. Il en résulte un sous-traitement.

- Dans ce questionnaire, nous souhaitons identifier les **compromis acceptables** entre :

La performance du test (pourcentage de sur-diagnostiqués / sous-diagnostiqués),

Le coût direct du test pour les femmes, et

Le délai de résultat.

* Le test de réaction en chaîne par polymérase (PCR) est un type de test d'acide nucléique.

** Les charges virales élevées sont définies comme les niveaux d'ADN du VHB ≥ 200 000 UI/mL.

**Section 2: Exemple**

Avant de répondre aux questions, imaginez que :

- Vous êtes un personnel de santé responsable des soins prénatals dans un centre de santé dans une zone rurale d'un pays à faible revenu. La plupart des gens sont très pauvres et vivent avec moins de 1,9 USD/jour.

- Chaque année, vous identifiez environ 1000 femmes infectées par le VHB dans vos services de dépistage prénatal.

Parmi ces femmes infectées par le VHB, environ 20 % (200 femmes) ont une charge virale élevée et ont besoin de médicaments antiviraux pour arrêter la transmission du VHB à leurs bébés.

Les autres (800 femmes) ont une charge virale basse et ne nécessitent pas de médicaments antiviraux.

Cependant, la plupart des femmes ne bénéficient pas des médicaments antiviraux parce qu'elles ne peuvent pas se permettre de supporter le coût de la PCR (40 US$) et de faire un voyage d'une journée dans un hôpital de district pour la PCR.

- Vous êtes maintenant interrogé sur votre préférence entre deux tests rapides fictifs comme outil de détection des femmes enceintes infectées par le VHB avec des charges virales élevées.


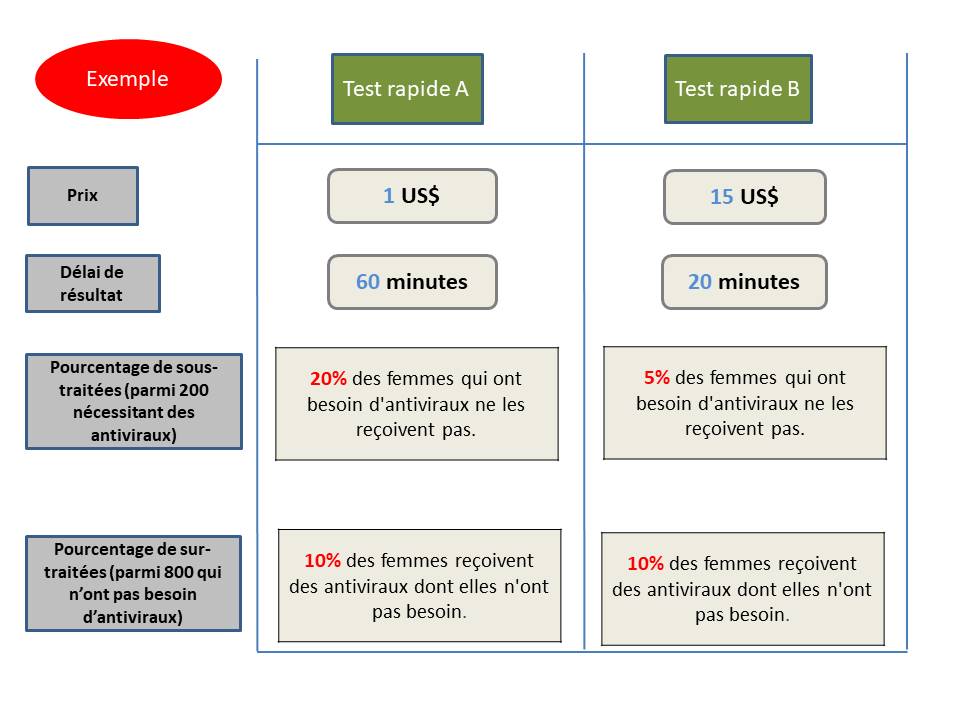


- Le test A est beaucoup plus abordable (US$ 1) que le test B (US$ 15) pour les femmes.

- Le délai d'obtention du résultat est plus long avec le test A (60 min) qu'avec le test B (20 min).

- Sur les 200 femmes qui ont besoin d'un traitement, 20 % ne seront pas détectées en utilisant le test A. En utilisant le test B, 5 % ne seront pas détectées.

- Sur 800 femmes qui n'ont pas besoin de traitement, 10 % seront traitées inutilement à l'aide du test A. Le même pourcentage de femmes seront traitées inutilement à l'aide du test B.

- Après avoir choisi l'un des tests rapides, il vous sera demandé si vous accepteriez ce test rapide plutôt que la PCR classique. Avec la PCR, il existe un risque de perte de suivi des femmes en raison du coût du test et des déplacements.

**Section 3: Le questionnaire**

Au début, vous aurez sept (7) scénarios dans lesquels il vous sera demandé de choisir entre deux tests rapides différents à chaque fois. Ensuite, on vous posera des questions sur votre pays et votre travail.

Merci d'avance de remplir ce questionnaire.

**Question sur les scénarios de tâche de choix**

1. Quel test rapide préférez-vous ?

Test rapide A

Test rapide B

2. Accepteriez-vous ce test rapide plutôt que la PCR* ?

OUI

NON

* En choisissant la PCR, la femme peut abandonner car elle doit faire un voyage d'une journée dans un hôpital de district et supporter le coût de la PCR (40 USD).

**** Si un participant répond NON à la question 2 pour toutes les tâches à choix,**

3. Pourriez-vous indiquer les principales raisons pour lesquelles vous n'accepteriez jamais un test rapide plutôt qu'un PCR ?

La PCR a des meilleures performances

Autre (veuillez préciser……………)

**Questions sur vous-même**

Q1 : Dans quelle classe d’âge êtes-vous?

< 30 ans

30-50 ans

> 50 ans

Q2 : Quel est votre sexe ?

Femme

Homme

Autre

Q3 : Dans quel pays travaillez-vous ?

………………………………

Q4 : Quelle est votre profession?

Médecin

Infirmière

Sage-femme

Personnel de laboratoire

Praticien en santé publique

Autre (veuillez préciser ________)

Q5 : Dans quel secteur travaillez-vous ?

Soins de santé dans le secteur public

Premiers soins

Hôpital de district

Hôpital provincial/régional

Hôpital national

Soins de santé dans le secteur privé

Autre (veuillez préciser _______)

Q6 : Êtes-vous impliqué dans les soins de l'hépatite ?

Non

Oui

**1C. Portuguese version**

**Seção 1: Introdução**

Convidamo-lo a participar nesta pesquisa online acerca da sua preferência sobre as características de um teste de diagnóstico rápido fictício para identificar mulheres grávidas infectadas com o vírus de hepatite B (HBV) com um elevado risco de transmissão de mãe para filho.

- Para complementar a dose da vacina contra a Hepatite B para a prevenção da transmissão do vírus da mãe para filho, a OMS recomenda:

Todas as mulheres grávidas devem ter um teste de rastreio de infecção de hepatite B.

Todas as mulheres positivas ao teste de rastreio de HBV devem ainda se submeter a um *teste de PCR** para quantificar as cargas virais da hepatite B.

Todas as mulheres com uma *carga viral elevada*** pelo teste de PCR deve receber medicamentos antivirais.

- Mas, em muitos países de baixos rendimentos, as mulheres infectadas pelo HBV NÃO beneficiam de medicamentos antivirais porque:

O teste de PCR NÃO está disponível em clínicas de cuidados primários

As mulheres devem pagar do seu próprio bolso e NÃO podem pagar o preço elevado dos testes de PCR (US$ > 40/teste)

- Por conseguinte, será útil fazer um teste rápido para distinguir entre cargas virais “*elevadas*” e “*baixas*”:

A um custo mais baixo acessível para as mulheres

Usando uma gota de sangue do dedo

Por funcionários não-laboratoriais (médicos, enfermeiros, parteiras) em clínicas de cuidados primários

Dentro de um curto período de tempo

- No entanto, a grande desvantagem de um teste rápido, os resultados podem não ser tão precisos como o teste PCR:

Com um risco de um diagnóstico-excessivo, identificar incorretamente as mulheres como tendo uma *carga viral elevada* quando a sua carga viral é *baixa*. Isto resulta em sobretratamento.

Com o risco de subdiagnóstico, identificando incorretamente as mulheres como tendo uma *carga viral baixa* quando a sua carga viral é *elevada*. Isso resulta em subtratamento.

- Neste questionário, gostaríamos de identificar **as soluções de compromisso aceitáveis** entre elas:

O desempenho do teste (percentagem de sobrediagnosticados / subdiagnosticados),

O custo extra dos testes para as mulheres, e

O tempo para entrega do resultado.

* O teste ensaio de reacção em cadeia da polimerase de PCR (*Polymerase Chain Reaction*) é um tipo de teste com ácido nucleico.

** As elevadas cargas virais são definidas como níveis de ADN HBV ≥ 200 000 IU/mL.

**Seção 2: Exemplo**

Antes de responder às questões, por favor imagine que:

- É um profissional de saúde responsável pelos cuidados pré-natais numa clínica de cuidados primários numa zona rural de um país de renda-baixa. A maioria das pessoas é muito pobre, vivendo abaixo de 1,9 dólares/dia.

- Em cada ano, identifica cerca de 1000 mulheres infectadas com HBV nos seus serviços de rastreio pré-natal.

Destas mulheres infectadas com HBV, cerca de 20% (200 mulheres) têm cargas virais elevadas e necessitam de medicamentos antivirais para impedir a transmissão do HBV aos seus bebés.

As restantes (800 mulheres) têm cargas virais baixas e não necessitam de medicamentos antivirais.

Contudo, a maioria das mulheres não se beneficia dos mediamentos antivirais porque não podem arcar o custo dos testes de PCR (40 dólares) e fazer viagem de um dia a um hospital distrital para se submeterem a um teste de PCR.

- É-lhe agora perguntado sobre a sua preferência entre dois testes rápidos fictícios como ferramenta para detectar mulheres grávidas infectadas com HBV com cargas virais elevadas.


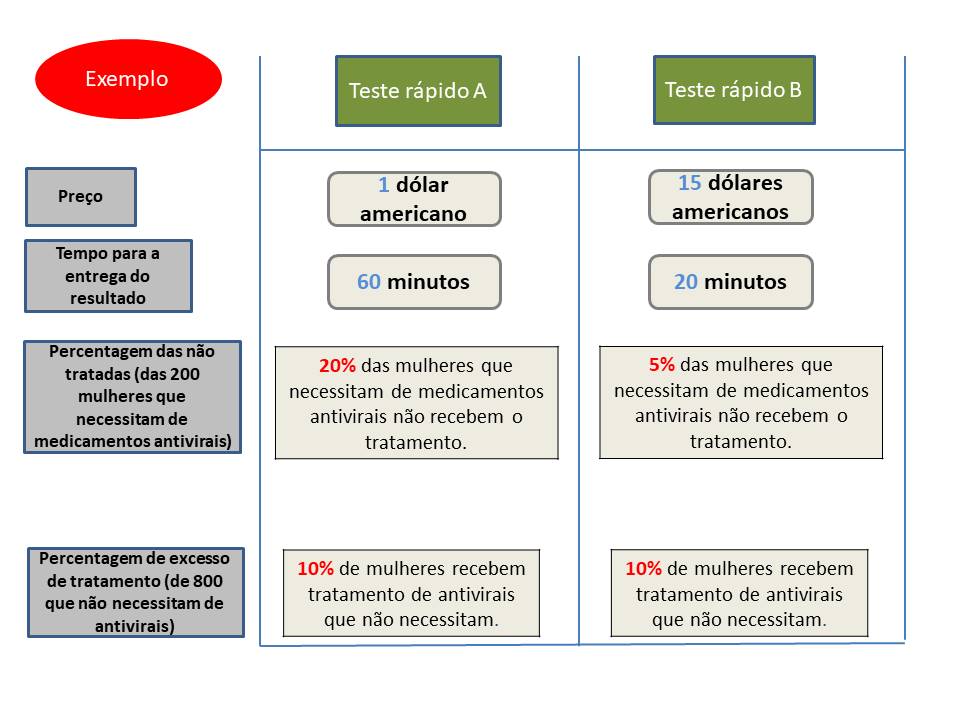


- O teste A é muito mais acessível (1 dólar) do que o teste B (15 dólares) para as mulheres.

- O tempo necessário para entrega do resultado é mais longo com o teste A (60 minutos) do que com o teste B (20 minutos).

- Das 200 mulheres que necessitam de tratamento, 20% perderão a oportunidade de utilizar o teste A. Ao utilizar o teste B, 5% não serão atendidas.

- Das 800 mulheres que não necessitam de tratamento, 10% serão tratadas desnecessariamente com o teste A. A mesma percentagem de mulheres será tratada desnecessariamente com o teste B.

- Depois de escolher um dos testes rápidos, ser-lhe-á perguntado se aceitaria este teste rápido em vez do PCR convencional. Com o PCR, existe o risco de se perder o acompanhamento das mulheres devido ao custo do teste e da viagem.

**Seção 3: O questionário**

No início, terá sete (7) cenários em que você será convidado a escolher entre dois testes rápidos diferentes em cada vez. Em seguida, ser-lhe-á perguntado sobre o seu país e o seu trabalho.

Agradecemos-lhe antecipadamente o preenchimento deste questionário.

**Pergunta sobre cenários de escolha de tarefas**

1. Qual é o teste rápido que prefere?

Teste rápido A

Teste rápido B

2. Você aceitaria este teste rápido ao invés do PCR*?

SIM

NÃO

* Ao escolher a PCR, a mulher pode desistir, pois tem de fazer uma viagem de um dia a um hospital distrital e suportar o custo da PCR (40 dólares).

**** Se um participante escolher NÃO na pergunta 2 para todas as escolhas das tarefas,**

3. Poderia indicar as principais razões pelas quais nunca aceitaria um teste rápido *ao invés de um teste de PCR*" ?

O PCR tem melhor desempenho

Outros (especificar……………)

**Pergunta sobre si mesmo**

Q1: Qual é a sua taxa etária?

< 30 anos de idade

30-50 anos de idade

> 50 anos de idade

Q2: Qual é o seu género?

Mulher

Homem

Outro

Q3: Em que país trabalha?

………………………………

Q4: Qual é a sua profissão?

Médico

Enfermeira/o

Parteira/obstetra

Pessoal de laboratório

Profissional de saúde pública

Outro (especificar ________)

Q5: Em que sector trabalha?

Cuidados de saúde no sector público

Cuidados primários

Hospital distrital

Hospital provincial/regional

Hospital nacional

Cuidados de saúde no sector privado

Outro (especificar _______)

Q6: Está envolvido no tratamento da hepatite?

Não

Sim
