# Supplementary material for: Informing a target product profile for rapid tests to identify HBV-infected pregnant women with high viral loads: a discrete choice experiment with African healthcare workers
Source: BMC Med. 2023 Jul 4;21:243. doi: 10.1186/s12916-023-02939-y (PMC10320875; doi:10.1186/s12916-023-02939-y)
Supplement: Supplementary file 2 — Additional file 2. Experimental design. [file 12916_2023_2939_MOESM2_ESM.docx]

**Additional file 2. Experimental design**

The cost levels of US$ 5, 15 and 20 were assigned prior β values of -0.1, -0.2, -0.4 respectively (compared to cost = US$ 1). The 60 minute time-to-result level was assigned β of -0.2 (compared to time-to-result = 20 minute). Sensitivity levels of 90%, 95% and 100% were assigned β of 0.6, 0.8 and 1 respectively (compared to sensitivity = 85%) while specificity levels of 95% and 100% had been assigned respective β values of 0.1 and 0.2 (compared to specificity = 90%). We verified that the resulting design using prior values of preference parameters β dominated the standard orthogonal design (with 0 prior values) in terms of efficiency and in particular, minimization of dominated or dominant test alternatives (i.e., tests with better levels for all attributes). We determined that a minimum of 12 pairs of choice set were needed in the DCE to estimate all main effects (12 is the lowest common denominator between all attributes’ levels numbers).
