# Supplementary material for: Informing a target product profile for rapid tests to identify HBV-infected pregnant women with high viral loads: a discrete choice experiment with African healthcare workers
Source: BMC Med. 2023 Jul 4;21:243. doi: 10.1186/s12916-023-02939-y (PMC10320875; doi:10.1186/s12916-023-02939-y)
Supplement: Supplementary file 3 — Additional file 3. Mixed multinomial logitmodel. [file 12916_2023_2939_MOESM3_ESM.docx]

**Additional file 3. Mixed multinomial logit (MIXL) model**

We used a mixed multinomial logit (MIXL) model, allowing the preference parameters to be randomly distributed across the sample and thus accounting for (i) unobserved preference heterogeneity and (ii) correlation of choices within participants (McFadden D & Train K, 2000). We specified the main utility function as follows:

*U_njt_* = *β*_1_,*_n_*Cost_2_*_j_* + *β*_2_,*_n_*Cost_3_*_j_* + *β*_3_,*_n_*Cost_4_*_j_* + *β*_4_,*_n_*Sensitivity_2_*_j_* + *β*_5_,*_n_*Sensitivity_3_*_j_* + *β*_6_,*_n_*Sensitivity_4_*_j_* + *β*_7_,*_n_*Specificity_2_*_j_* + *β*_8_,*_n_*Specificity_3_*_j_* + *β*_9_,*_n_*Time_2_*_j_* *+ ε_njt_*

where *U*_njt_ is the utility an individual *n* derives from choosing alternative (scenario) *j* in choice situation *t*, *Cost*_2*_j_*,…, *Time*_2*_j_* represent dummy coded attributes’ levels displayed in scenario *j* (level 1 of all attributes was used as reference), *β* _1,_*_n_*,…, *β* _9,_*_n_* are the respective random effects (part-worth utilities) associated with each attribute level (compared with the reference), the subscript *n* denoting respondent-specific parameters. Finally, *ε_njt_* is the error term assumed extreme value type 1 distributed, thus leading to the multinomial logit choice specification (McFadden D, 1974). We estimated a MIXL with correlated random coefficients between all nine attributes’ levels assuming normal distribution for each parameter *β_k_*_,_*_n._* Though particularly computationally intensive, this model is known to be most flexible (Hess S & Train K, 2017), thus allowing to account for correlation patterns between unobserved determinants of choice as well as various degrees of choice consistency across respondents (Hess S & Rose JM, 2012).

In the MIXL model with continuous attributes, we added an alternative specific constant (ASC) for test A (ASC_testA_) with *α_n_* the associated random coefficient representing the propensity to select test A vs. test B, irrespectively of attributes’ levels values.

*U_njt_* = α*_n_ASC*_test_*_A_* + *β*Cost + *β*Sensitivity + *β*Specificity + *β*Time *+ ε_njt_*
