# Supplementary material for: Informing a target product profile for rapid tests to identify HBV-infected pregnant women with high viral loads: a discrete choice experiment with African healthcare workers
Source: BMC Med. 2023 Jul 4;21:243. doi: 10.1186/s12916-023-02939-y (PMC10320875; doi:10.1186/s12916-023-02939-y)
Supplement: Supplementary file 4 — Additional file 4: Table S1. Description of choice tasks and choice pattern between rational and non-rational responders. Table S2. Alternative-specific multinomial probit regression model with categorical attribute levels. Table S3. Alternative-specific multinomial probit regression model with continuous attribute levels. Table S4. Mixed multinomial logitmodel with categorical attribute levels. Table S5. Minimal and optimal target product profile. Figure S1. Scatter plots showing the correlation between the levels of cost, sensitivity and specificity, and the corresponding values for the utility in the rational responders. [file 12916_2023_2939_MOESM4_ESM.docx]

**Additional file 4. Tables & figures**

**Table S1. Description of choice tasks and choice pattern between rational and non-rational responders**

| **Choice task** | **Test A** | | | |  | **Test B** | | | |  | **Participants choosing Test A** | | | |
| --- | --- | --- | --- | --- | --- | --- | --- | --- | --- | --- | --- | --- | --- | --- |
|  | Cost | Sensitivity | Specificity | Time |  | Cost | Sensitivity | Specificity | Time |  | Total, n (%) | Non-rational respondents, n (%) | Rational respondents, n (%) | P-value |
| **Block 1** |  |  |  |  |  |  |  |  |  |  |  |  |  |  |
| 1 | 10 USD | 95% | 95% | 60 mins |  | 1 USD | 90% | 90% | 20 mins |  | 129 (47.8) | 26 (65.0) | 103 (44.8) | 0.018 |
| 2 | 20 USD | 100% | 95% | 20 mins |  | 5 USD | 90% | 100% | 60 mins |  | 136 (50.4) | 21 (52.5) | 115 (50.0) | 0.770 |
| 3 | 1 USD | 95% | 95% | 60 mins |  | 5 USD | 85% | 100% | 20 mins |  | 225 (83.3) | 31 (77.5) | 194 (84.3) | 0.283 |
| 4 | 20 USD | 95% | 90% | 60 mins |  | 10 USD | 100% | 100% | 20 mins |  | 40 (14.8) | 40 (100.0) | 0 (0.0) | <0.001 |
| 5 | 10 USD | 95% | 90% | 20 mins |  | 5 USD | 100% | 95% | 60 mins |  | 44 (16.3) | 30 (75.0) | 14 (6.1) | <0.001 |
| 6 | 10 USD | 85% | 95% | 60 mins |  | 20 USD | 100% | 100% | 20 mins |  | 80 (29.6) | 28 (70.0) | 52 (22.6) | <0.001 |
| 7 | 10 USD | 90% | 100% | 20 mins |  | 1 USD | 85% | 90% | 60 mins |  | 185 (68.5) | 28 (70.0) | 157 (68.3) | 0.827 |
| **Block 2** |  |  |  |  |  |  |  |  |  |  |  |  |  |  |
| 8 | 1 USD | 85% | 100% | 20 mins |  | 10 USD | 90% | 90% | 60 mins |  | 216 (75.8) | 31 (81.6) | 185 (74.9) | 0.371 |
| 9 | 20 USD | 90% | 100% | 60 mins |  | 5 USD | 100% | 90% | 20 mins |  | 51 (17.9) | 17 (44.7) | 34 (13.8) | <0.001 |
| 10 | 5 USD | 90% | 90% | 60 mins |  | 10 USD | 85% | 100% | 20 mins |  | 191 (67.0) | 26 (68.4) | 165 (66.8) | 0.843 |
| 11 | 20 USD | 90% | 95% | 60 mins |  | 1 USD | 95% | 100% | 20 mins |  | 38 (13.3) | 38 (100.0) | 0 (0.0) | <0.001 |
| 12 | 20 USD | 100% | 100% | 60 mins |  | 1 USD | 90% | 95% | 20 mins |  | 152 (53.3) | 30 (79.0) | 122 (49.4) | 0.001 |
| 13 | 5 USD | 95% | 95% | 20 mins |  | 10 USD | 100% | 90% | 60 mins |  | 183 (64.2) | 27 (71.0) | 156 (63.2) | 0.345 |
| 14 | 1 USD | 90% | 95% | 60 mins |  | 5 USD | 95% | 90% | 20 mins |  | 129 (45.3) | 25 (65.8) | 104 (42.1) | 0.006 |

|  | Better level |
| --- | --- |
|  | Worse level |
|  | All levels are better |
|  | All levels are worse |

Table shows the two blocks of seven choice tasks with two fictional RDTs (tests A and B), representing a total of 14 choice tasks. The first block contains choice tasks 1-7, and the second block contains choice tasks 8-14. Choice task 4 in block 1 and choice task 11 in block 2 are dominant scenarios, in which all the attribute levels of test B are better than those of test A. To investigate whether the pattern of choices differed between rational and non-rational responders, we compared the proportion that chose test A over test B in each choice task between these two groups using chi-squared test. After excluding two dominant choice tasks, in 75% (8/12) of choice tasks the difference was statistically significant (p < 0.05).

**Table S2. Alternative-specific multinomial (ASM) probit regression model with categorical attribute levels**

| Alternative-specific multinomial (ASM) probit regression | | | | | | | |
| --- | --- | --- | --- | --- | --- | --- | --- |
|  | Full sample (n=555) | | |  | Rational responders (n=477) | | |
| Attribute levels | Coefficient | Std. err. | P-value |  | Coefficient | Std. err. | P-value |
| Cost US$ 5 | -0.186 | 0.064 | 0.004 |  | -0.141 | 0.071 | 0.046 |
| Cost US$ 15 | -0.706 | 0.058 | <0.001 |  | -0.809 | 0.063 | <0.001 |
| Cost US$ 20 | -1.118 | 0.096 | <0.001 |  | -1.293 | 0.107 | <0.001 |
| Sensitivity 90% | 0.527 | 0.056 | <0.001 |  | 0.621 | 0.062 | <0.001 |
| Sensitivity 95% | 1.064 | 0.080 | <0.001 |  | 1.124 | 0.089 | <0.001 |
| Sensitivity 100% | 1.461 | 0.083 | <0.001 |  | 1.763 | 0.094 | <0.001 |
| Specificity 95% | 0.403 | 0.048 | <0.001 |  | 0.470 | 0.055 | <0.001 |
| Specificity 100% | 0.522 | 0.054 | <0.001 |  | 0.579 | 0.060 | <0.001 |
| Time 60 min | -0.031 | 0.035 | 0.375 |  | -0.058 | 0.038 | 0.131 |

**Table S3. Alternative-specific multinomial (ASM) probit regression model with continuous attribute levels**

| Alternative-specific multinomial (ASM) probit regression | | | | | | | |
| --- | --- | --- | --- | --- | --- | --- | --- |
|  | Full sample (n=555) | | |  | Rational responders (n=477) | | |
| Attributes | Coefficient for unit increase | Std. err. | P-value |  | Coefficient for unit increase | Std. err. | P-value |
| Cost | -0.056 | 0.003 | <0.001 |  | -0.068 | 0.004 | <0.001 |
| Sensitivity | 0.094 | 0.005 | <0.001 |  | 0.113 | 0.005 | <0.001 |
| Specificity | 0.050 | 0.005 | <0.001 |  | 0.058 | 0.006 | <0.001 |
| Time | -0.013 | 0.033 | 0.700 |  | -0.027 | 0.036 | 0.453 |

**Table S4. Mixed multinomial logit (MIXL) model with categorical attribute levels**

| MIXL model with categorical attribute levels | | | |
| --- | --- | --- | --- |
|  | Rational responders (n=477) | | |
| Attribute levels | Coefficient | Standard Error | P-value |
| Cost US$ 5 | -0.223 | 0.119 | 0.061 |
| Cost US$ 15 | -1.571 | 0.137 | <0.001 |
| Cost US$ 20 | -2.550 | 0.287 | <0.001 |
| Sensitivity 90% | 1.341 | 0.134 | <0.001 |
| Sensitivity 95% | 2.343 | 0.212 | <0.001 |
| Sensitivity 100% | 3.749 | 0.298 | <0.001 |
| Specificity 95% | 1.090 | 0.176 | <0.001 |
| Specificity 100% | 1.134 | 0.130 | <0.001 |
| Time 60 min | -0.284 | 0.084 | 0.001 |

**Table S5. Minimal and optimal target product profile (TPP)**

|  | Minimal and optimal TPP at a fixed specificity of 95% | | | | | |
| --- | --- | --- | --- | --- | --- | --- |
| Time-to-result | | 20 mins | |  | 60 mins | |
| TPP |  | Sensitivity | |  | Sensitivity | |
|  |  | Minimal | Optimal |  | Minimal | Optimal |
| Cost | US$ 1 | 82.5 | 87.5 |  | 83.5 | 88.0 |
|  | US$ 5 | 85.0 | 90.0 |  | 85.5 | 90.5 |
|  | US$ 15 | 90.5 | 95.5 |  | 91.5 | 96.0 |
|  | US$ 20 | 93.5 | 98.5 |  | 94.0 | 99.0 |
|  | Minimal and optimal TPP at a fixed specificity of 90% | | | | | |
| Time-to-result | | 20 mins | |  | 60 mins | |
| TPP |  | Sensitivity | |  | Sensitivity | |
|  |  | Minimal | Optimal |  | Minimal | Optimal |
| Cost | US$ 1 | 85.0 | 90.0 |  | 85.5 | 91.5 |
|  | US$ 5 | 87.5 | 93.5 |  | 88.0 | 93.0 |
|  | US$ 15 | 93.0 | 98.0 |  | 93.5 | 98.5 |
|  | US$ 20 | 96.0 | 100.0 |  | 96.5 | 100.0 |

**Figure S1. Scatter plots showing the correlation between the levels of cost, sensitivity and specificity, and the corresponding values for the utility in the rational responders (n=477)**
